# Density and Coexistence Patterns of an Apex Carnivore (*Panthera pardus*) and a Mesocarnivore (*Caracal aurata*) in Northern Congo Forests

**DOI:** 10.3390/ani16020190

**Published:** 2026-01-08

**Authors:** Sarah Tossens, Zoe Woodgate, Jean-Louis Doucet, Philipp Henschel, Adrien André, Johan Michaux, Marine Drouilly

**Affiliations:** 1 Gembloux Agro-Bio Tech, Forest Is Life, Université de Liège, Passage des Déportés 2, 5030 Gembloux, Belgium; 2 Panthera, West 40th Street, 18th Floor, New York, NY 10018, USA; 3 Institute for Communities and Wildlife in Africa, University of Cape Town, HW Pearson Building, University Avenue North, Rondebosch, Cape Town 7701, South Africa; 4 GeCoLAB, Université de Liège, Chemin de la Vallée 4, 4000 Liège, Belgium; 5 Centre for Social Science Research, University of Cape Town, Robert Leslie Social Science Building, 12 University Avenue South, Rondebosch, Cape Town 7701, South Africa

**Keywords:** Central Africa, competition, diet, golden cat, intraguild interactions, leopard, multispecies occupancy, niche partitioning, spatiotemporal patterns, SCR

## Abstract

Understanding how carnivores coexist is central to ecological theory and conservation. Coexistence among sympatric species arises through niche partitioning across spatial, temporal, and trophic dimensions, yet these mechanisms remain poorly explored in Central African forests where leopards (*Panthera pardus*) and African golden cats (*Caracal aurata*) act as dominant and subordinate carnivores. Using camera trap data and molecular scat analyses from two sites in northern Congo, we provided the first robust leopard density estimates for the region (i.e., semideciduous forests in Central Africa) and assessed coexistence mechanisms between the two felids across spatial, temporal, and trophic axes. Spatially explicit capture–recapture models revealed comparable leopard densities across sites (5–6 individuals/100 km^2^), exceeding the regional average for Central and East Africa. Spatiotemporal occupancy models indicated spatial and temporal overlap, with no evidence of predictive or reactive temporal avoidance, though fine-scale co-occurrence declined near linear forest features (i.e., main rivers and roads) where both species’ marginal occupancy was highest. Conversely, dietary analyses showed trophic segregation: leopards consumed medium- to large-sized ungulates (>20 kg), whereas golden cats relied on smaller prey (≤5 kg), identifying trophic partitioning as the main axis facilitating coexistence in this prey-rich system. Maintaining prey diversity and minimizing disturbance are key to sustaining both species and their coexistence mechanisms. Such multidimensional approaches are essential to understand intraguild interactions and anticipate community shifts under increasing pressure.

## Simple Summary

Carnivores interact with one another and reduce competition by partitioning space, time, and/or food. In Central African rainforests, leopards are the dominant carnivore, while African golden cats are a subordinate medium-sized carnivore. Until now, little was known about how these two species manage to coexist. Using camera traps and genetic scat analyses from two sites in northern Congo, this study provided the first leopard density estimates for the region and examined how these two wild cats share space, time, and food. Leopards were found at relatively high densities compared to other regions of Central and East Africa. The two species often used the same areas and were active at similar times, showing little evidence of avoiding each other. However, along rivers and roads where both species were most common, they were less likely to be detected together, suggesting some fine-scale avoidance. In contrast, their diets differed significantly: leopards mainly consumed larger animals (>20 kg), such as red river hogs, while golden cats fed on smaller prey (≤5 kg), like rodents. This dietary difference appears to be the main way they reduce competition. Maintaining diverse prey and minimizing human disturbance are essential to maintaining both species and the ecological balance they support.

## 1. Introduction

Terrestrial ecosystems support diverse carnivore assemblages that play central roles in shaping community structure and dynamics [1,2]. These assemblages typically comprise a spectrum of large and smaller carnivores whose functional roles and competitive hierarchies vary with body size and trophic position [3,4]. Large carnivores, generally acting as apex carnivores (i.e., dominant), have long been recognized as key drivers of ecosystem processes through their interactions with prey and competitors, sometimes triggering cascading effects on lower trophic levels [5,6]. More recently, it has been recognized that medium- and small-sized carnivores (<20 kg), often referred to as mesocarnivores (i.e., subordinate), also fulfill significant ecological functions, ranging from predation on small prey to scavenging and seed dispersal [7,8]. Inter- and intra-specific coexistence within such guilds may emerge from a balance between exploitative competition, driven by shared resource use [9,10], interference competition, such as fear-mediated behaviors and direct antagonism, including intraguild predation [11,12,13], and facilitative interactions, where one species indirectly benefits another [4,14]. These processes, reflecting the behavioral plasticity of carnivores, can occur at broad scales, for instance when mesocarnivores shift activity to spatial or temporal refuges less frequented by apex carnivores [15,16], or at fine scales, through short-term adjustments of avoidance or attraction [17,18].

Diverse carnivore guilds persist largely through mechanisms of niche partitioning that enable coexistence among sympatric species across spatial, temporal, and trophic axes [19,20]. Competition-centered niche theory predicts that species competing for similar resources must segregate, at least partially, along one or more of these three ecological niche dimensions [19,21]. However, few studies have evaluated all three axes simultaneously (but see [22,23]). Adopting a multidimensional perspective is therefore critical to identify the mechanisms enabling carnivore coexistence [24,25].

These partitioning patterns are highly context-dependent [5,26]. Carnivore abundance, behaviors, and intraguild interactions are strongly modulated by environmental and anthropogenic conditions that shape resource distribution, habitat structure, and disturbance regimes [27,28,29]. For instance, prey-rich areas may relax competitive pressures, allowing greater spatiotemporal overlap between dominant and subordinate carnivores [30]. Regions under high anthropogenic disturbance can promote niche partitioning by releasing or creating additional niche space along one or more axes [31]. Along the trophic axis, for example, anthropophilic species may exploit alternative food sources, such as livestock, thereby mitigating competition with dominant competitors (e.g., [32,33]). Alternatively, competing species may instead be constrained to co-occur in limited refuges due to their shared avoidance of humans, thereby intensifying competitive interactions (e.g., [34,35]). In such cases, coexistence may be less governed by direct competition than by shared responses to resources and disturbance [36,37].

Despite some recent range expansions, particularly in the Northern Hemisphere, where most carnivore coexistence studies have been conducted [26,38], most carnivore species are in decline, and remain conservation priorities [39,40]. This dual context highlights the importance of advancing our understanding of carnivore community dynamics in ecosystems that are both highly threatened and understudied, to inform conservation and management strategies [28,41].

The rainforests in the northern Republic of Congo represent such a system, where leopards (*Panthera pardus*; ABM = 43 kg) and African golden cats (*Caracal aurata*; ABM = 9 kg) coexist as the apex carnivore and the largest mesocarnivore, respectively, both exerting dominance over a smaller carnivore guild (e.g., African Palm civets (*Nandinia binotata*), genets, and mongooses) [42]. Although the presumed ranges of both felids overlap from West African forests of Senegal to East African forests of Uganda, their distributions are increasingly fragmented and reduced, with the golden cat being strictly forest-dependent [43,44]. Despite the growing threats they face, both species remain poorly studied, with the golden cat still considered to be one of the least known small felids worldwide [44]. Across their range, the paucity of published research on the mechanisms facilitating their coexistence highlights a critical gap in our ecological and conservation knowledge (but see [45,46]). Understanding how carnivore interactions unfold in tropical forests is critical, as biotic interactions play a major role in shaping biodiversity and the functioning of biotic communities [47,48]. In carnivore guilds, such dynamics can cascade through food webs, influencing mesopredator control and prey populations, with potential far-reaching ecological consequences [3,6]. This makes the study of coexistence mechanisms highly relevant for both theory and applied conservation [15,49].

In this context, we pursued two main objectives. First, we aimed to estimate the density of both species in two forested sites of northern Congo to provide baseline information on this apex carnivore–mesocarnivore system. Second, we examined comprehensively, for the first time, how leopards and African golden cats partition their ecological niches across spatial, temporal, and trophic axes. We hypothesized that (H1) golden cats would spatially avoid leopards (i.e., lower site-use probability and reduced co-occurrence), (H2) golden cats would temporally avoid leopards where they spatially co-occur (i.e., reduced temporal overlap and fine-scale avoidance reflected by reduced detection following leopard detections), reflecting interference risk [22,50], and (H3) golden cats and leopards would exhibit dietary segregation (i.e., low trophic overlap and contrasting prey size classes) linked to body-size differences and associated prey size preferences [44,45]. Furthermore, we tested whether co-occurrence patterns varied with the availability of leopards’ preferred local prey (i.e., the red river hog, *Potamochoerus porcus*), as prey-rich areas could reduce competition for shared prey resources and thus facilitate coexistence [30], and with proximity to linear forest features (i.e., main river or road), as such structural elements and potential associated anthropogenic disturbance can alter carnivore behavior and interactions [51]. To test these hypotheses, we used camera trap data to investigate spatial, temporal, and fine-scale interactions between the species [52,53], and molecular analyses of non-invasively collected scats to assess diet composition, niche breadth, and diet partitioning of these two sympatric felids [54,55].

## 2. Materials and Methods

### 2.1. Study Areas

**Figure 1:**
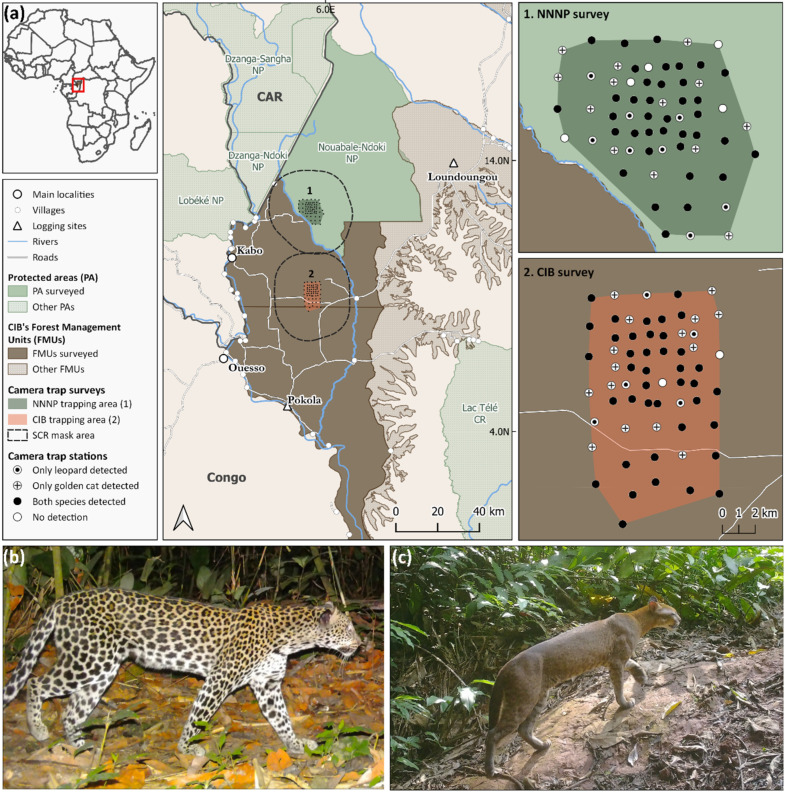
(**a**) Map of both study sites in northern Republic of Congo, showing the Nouabalé-Ndoki National Park (NNNP) and Forest Management Units (FMUs) of the logging company Congolaise Industrielle des Bois (CIB). Survey areas are outlined in dark green and orange. Right panels illustrate grid design, consisting of a nested structure with an internal 1 × 1 km grid and a larger 2 × 2 km grid, as well as the location of camera trap stations categorized by species detections. Photographs of a leopard (**b**) and a golden cat (**c**), captured on a wildlife trail in dense terra firma forest.

The study was conducted in the Nouabalé-Ndoki National Park (NNNP) (2°14′ N, 16°25′ E), which spans over 4300 km^2^ of contiguous lowland rainforests in the northern Republic of Congo, and its buffer zone, a forest concession granted to the logging company Congolaise Industrielle des Bois (CIB) (Ouesso, Republic of Congo; a subsidiary of Olam-Agri) (1°55′ N, 16°25′ E), covering over 21,000 km^2^ (Figure 1). Established in 1993, NNNP encompasses pristine tropical forest that is protected from hunting and has been inscribed as a UNESCO World Heritage Site since 2012, as part of the Sangha Trinational Network of protected areas (TNS) [56]. In 1997, CIB obtained concessionary rights to three continuous Forest Management Units (FMUs; ‘Kabo’, ‘Pokola’, and ‘Loundoungou-Toukoulaka’) adjacent to the park for selective timber extraction, which have been certified by the Forest Stewardship Council (FSC) since 2006. In 1999, both areas were linked through the PROGEPP (Ecosystem Management Project for the Periphery of the Nouabalé-Ndoki National Park), a tripartite partnership between a conservation organization (Wildlife Conservation Society), a private company and the public sector, to strengthen wildlife conservation efforts and sustainable management in the Ndoki-Likouala landscape [57].

The study region is defined as Moist Central Africa [58], which is characterized by semideciduous and evergreen-semideciduous forests on sandstone [59]. It consists of mixed terra firma forests with low topographic relief, including Maranthaceae, dense, and secondary forest types dominated by *Fabaceae*, *Annonaceae* and *Malvaceae*, interspersed with swamp and monodominant *Gilbertiodendron* forests along watercourses [59,60]. Annual rainfall (mean = 1728 mm) is bimodal, peaking between August and November, and April and June. The Ndoki River is the largest in the study area, and serves as the border between NNNP and ‘Kabo’ FMU. This remote forested region is known to be an important refuge for several globally threatened large mammals, including the Critically Endangered forest elephants (*Loxodonta cyclotis*) [61] and western lowland gorillas (*Gorilla gorilla*) [56]. These forests also support a highly diverse range of medium- and small-sized species, providing a rich prey base for both leopards and golden cats [62] (see File S1 for detection rates of captured species).

### 2.2. Camera Trap Surveys

We conducted camera trap surveys in the southern sector of NNNP and the adjacent Kabo FMU over four- and six-month periods, respectively, between August 2022 and February 2023. Each survey site featured a grid of 63 paired camera trap stations. Grid size and camera trap spacing were designed considering species-specific monitoring guidelines for golden cats [44] and leopards [63] in comparable habitats.

Stations were arranged in a nested design: a 1 km sub-grid covering 36 km^2^ to meet golden cat survey requirements, embedded within a broader 2 km grid spanning approximately 144 km^2^—an area deemed sufficient to account for leopards’ ranging patterns (Figure 1).

In the logging concession, the grid location was chosen in consultation with concession managers based on three criteria: (i) the area had not yet been logged by CIB, reducing the influence of short-term logging impacts, (ii) it featured relatively high densities of wildlife signs, increasing the likelihood of detecting focal wild cats, known to avoid heavily disturbed areas [64,65], and (iii) it was logistically and physically accessible for field operations.

Camera trap stations were deployed within an average 200 m radius of grid cell centroids, preferentially along wildlife trails marked by signs of golden cats or leopards (e.g., spoor, scat, or scratch mark), which felids are known to favor for movement in tropical forests [66,67]. Roads were deliberately excluded to minimize the risk of camera theft. Each station comprised a pair of white-flash cameras (Bolyguard SG2060-D; Boly Inc., Shenzhen, China), mounted 40 cm above ground on opposite sides of the trail and slightly offset to capture both flanks of passing individuals while minimizing glare [68]. All cameras were set to take one photograph per trigger.

### 2.3. Density

In our study area, golden cats exhibited an unexpectedly low degree of coat pattern variability, presenting fewer spotted individuals than those photographed in Gabon (Figure 1c), where density estimation via spatially explicit capture–recapture (SCR) had previously been conducted [64]. This limited distinctiveness, combined with the scarcity of additional identifiable marks (e.g., scars, ear notches), further constrained our ability to distinguish individuals reliably in our dataset. Consequently, we were unable to proceed with SCR analyses for this species, as misidentification would introduce significant bias.

In contrast, leopards were reliably identified and sexed based on photographs, using visual inspection of external genitalia, dewlap size and unique pelage patterns. Both left and right flanks were used for the identification of males and females. Individuals that could not be confidently identified (i.e., fewer than three matching coat pattern features across independent camera trap images), as well as non-independent cubs and subadults, were excluded from analyses.

Adult leopard population density was estimated in each survey area using a maximum-likelihood SCR framework, implemented in the secr R package v. 5.2.1 [69] in R v. 4.4.1 [70]. The size of the state space (i.e., mask) was defined using a buffer equal to four to five times the root-pooled spatial variance (RPSV) around the trap array [68]. To meet the population closure assumption inherent to SCR analyses [71], we tested for demographic closure within each dataset using the closure test of Otis et al. (1978) [72]. As a result, we restricted each survey duration to the longest continuous sampling period during which closure could not be rejected (*p* > 0.05), thereby satisfying model assumptions.

Individual detection histories were assumed to follow a Bernoulli encounter process, with detection probability described using a half-normal detection function characterized by two parameters: *g*0 (i.e., individual capture probability at the activity center) and *σ* (i.e., spatial decay parameter) [71]. To test for sex-specific variation in both parameters, we fitted a null model alongside three models including sex as a covariate on *g*0, *σ*, or both. Models were ranked by Akaike’s Information Criterion (AIC; [73]) and the model with the lowest AIC value was considered the best supported by the data. To ensure mask size adequacy, we incrementally increased the buffer radius in 1 km steps and re-ran the top-ranked models to confirm that density estimates had stabilized [68].

We tested for departures from a 1:1 sex ratio at each site using a likelihood ratio test, comparing the best-performing model (with the mixing parameter *pmix* varying by sex) to an equivalent model with *pmix* fixed at 0.5. A non-significant result (*p* > 0.05) was interpreted as no deviation from parity in the observed sex ratio.

To assess the sensitivity of our density estimates, we computed the half relative confidence interval width (HRCIW) for each survey [74]. This metric quantifies the statistical power to detect potential population fluctuations over time. An HRCIW ≤ 50% is typically considered sufficient to detect a 50% decline in population size, meeting the IUCN Red List criterion A2 threshold for classifying a population as Endangered if such a decline occurs within ten years or three generations [68,74]. Higher-magnitude declines (e.g., ≥80%, Critically Endangered) would also be detectable with such precision.

### 2.4. Spatiotemporal Niche Partitioning

To explore spatial and temporal interactions between leopards and golden cats, while accounting for imperfect detection, we applied a multispecies occupancy model using a continuous-time detection process [75]. This approach extends traditional single-season occupancy models [76] by enabling joint inference on co-occurrence and temporal activity patterns, and by making full use of time-stamped camera trap detections. Detections of either species at each station were modeled as the outcome of a temporal Poisson point process, with detection intensity (λ(*t*)) varying as a function of time (*t*) and species-specific covariates.

The model estimates latent occupancy states across all species combinations using ‘natural parameters’ to represent the log-odds of presence for each species independently (first-order), and in combination with others (second- or higher-order; [77]). Species were assumed to occur independently when all second- and higher-order parameters were set to zero. These natural parameters can be modeled as linear functions of environmental covariates [52]. In the two-species case considered here, the first-order parameters (*f*_1_ and *f*_2_) describe the log-odds of station occupancy for golden cats and leopards, respectively, in the absence of one another. The second-order parameter (*f*_12_) represents the interaction effect between both species, capturing the change in log-odds of both species occurring at a station [75].

Occupancy probability was modeled as a function of site-specific covariates selected for their ecological relevance in the study system. These included a site effect accounting for potential variation across survey sites, distance to linear forest features (i.e., distance to the nearest linear element interrupting continuous forest cover, such as a major river or road), and the relative abundance index (i.e., RAI, number of independent capture events per 100 camera trap days) of leopards’ locally preferred prey (red river hog, *Potamochoerus porcus*; see ‘Trophic overlap’ section). In our study system, linear forest features consisted of the Ndoki River in NNNP and a permanent logging road within the CIB concession (Figure 1). Beyond representing structural breaks within the forest, these features may also function as potential human access routes into otherwise remote forest areas, thereby integrating both ecological and anthropogenic gradients likely to influence carnivore space use. Camera trap stations were located at varying distances from linear forest features (mean = 4.6 m, range = 0–10.9 m; see File S2 for the distribution of stations along the distance gradient).

We allowed prey availability to influence leopard occurrence (*f*_2_) and the probability of co-occurrence with golden cats (*f*_12_), while site effect and distance to forest edge were included across all natural parameters (*f*_1_, *f*_2_, and *f*_12_).

To characterize species-specific diel activity patterns, we modeled detection intensity using Fourier series of time-of-day, with two harmonics over a 24 h period, capturing the cyclical nature of daily activity rhythms [75]. Once the best-performing model for spatial (occupancy) predictors was identified, we incorporated a fifth covariate on golden cat detection intensity: the elapsed time since the last leopard detection, to test for fine-scale temporal interactions. Detection integrals were approximated using quadrature with 60 min intervals. All covariates were scaled and centered, and we verified that no pairwise correlation exceeded 0.6.

Competing models were ranked using AIC, with the model having the lowest AIC selected for parameter estimation. Parameters were considered statistically informative when their 95% confidence intervals (CIs) did not overlap zero. All models were fitted in R v. 4.4.1, using the ‘optim’ function [70] and a custom log-likelihood function developed in C++ by Kellner et al. (2022) [75].

### 2.5. Scat Collection and Diet Analyses

Scat samples of both felid species were collected opportunistically near camera trap stations and along trails during grid installation or retrieval at both study sites. This approach minimized spatiotemporal bias in scat sampling, ensuring comparable effort across sites. In the field, scats were pre-identified by food remains (e.g., hair) and morphological traits (e.g., diameter, characteristic ‘pearl necklace’ shape). From each, small portions of both surface and internal material were sampled. Because carnivore scats can be misidentified in the field [55,78], species origin was confirmed through molecular analysis. Each sample received a unique identifier and a standardized field record including sample ID, date, collector’s name, GPS coordinates, and contextual notes. To prevent DNA contamination, samples were collected using sterile techniques and preserved in sterile silica gel at ambient temperature until DNA extraction.

Species origin and diet were assessed using metabarcoding to PCR-amplify a 133 bp fragment of the mitochondrial Cytochrome C oxidase subunit I (CO1) gene with a universal vertebrate primer set adapted from Galan et al. (2018) [79]. This marker was chosen for broad taxonomic coverage and compatibility with reference databases, such as the Barcode of Life (BOLD) [80] and NCBI GenBank. Amplification was followed by a purification step, a secondary PCR for sample indexing, and a secondary purification step. Sequencing was performed on an Illumina NovaSeq^®^ flow cell (Illumina, Inc., San Diego, CA, USA) at the University of Liège GIGA Genomics platform. Sequence data were processed with an in-house bioinformatics pipeline adapted from André et al. (2017) [81], with taxonomic assignment based on sequence similarity to the BOLD and the NCBI GenBank. Details on filtering and assignment criteria can be found in File S3. Only samples with confirmed predator and prey identification were retained.

Diet composition was quantified using frequency of occurrence (FO, proportion of scats containing a prey category) and corrected FO (CFO, adjusted for multiple prey items per scat) [82]. To estimate relative biomass consumed per prey item (RBC), we applied the asymptotic regression model developed by Chakrabarti et al. (2016) [83] for obligate carnivores, adapted for leopards and golden cats. Adult body masses for leopards and golden cats were obtained from Castelló (2020) [42], and for prey species from Kingdon (2015) [84]. Prey were assigned to taxonomic groups (i.e., ungulates, primates, carnivores, rodents, birds) and to body mass classes based on species’ mean adult body mass across sexes: large (≥30 kg), medium (10–30 kg), small (2–10 kg), very small (≤2 kg) [85].

Trophic niche breadth was calculated in R v. 4.4.1 [70] for each predator using Levin’s standardized index [86] and dietary overlap using Pianka’s index (O; [87]). Finally, we evaluated the adequacy of scat sampling effort with the Brillouin index [88], using bootstrapped accumulation curves to determine whether prey diversity estimates had reached an asymptote [22,89]. Full formulas and computational details are provided in File S3.

## 3. Results

Across the two study sites, a total sampling effort of 34,916 camera trap nights across 125 stations yielded 367 detections (i.e., photographs taken > 1 min apart) of golden cats and 456 of leopards. Specifically for golden cats, we recorded 152 and 175 independent captures (i.e., photographs taken > 30 min apart) in NNNP and CIB, respectively. Leopards were independently photographed 186 times in NNNP and 179 times in CIB. Capture rates were highest in NNNP for both species (Table 1).

Overall, 61.6% of stations detected both felid species, compared to 32.8% that captured only one of them, and 5.6% that detected neither. The naive occupancy (i.e., proportion of stations with at least one detection) was slightly higher for golden cats (0.84) than for leopards (0.72) (Table 1).

### 3.1. Leopard Individual Identification

Out of 841 leopard photographs, 96.7% were suitable for identification. To meet the population closure assumption, we restricted the dataset to 110 survey days in NNNP (z-score = −1.64, *p* = 0.051) and 198 days in CIB (z-score = −1.61, *p* = 0.054), resulting in 162 and 168 unique leopard capture events, respectively. From these, we identified 18 individuals in NNNP (7 males, 7 females, 4 unknown sex) and 22 individuals in CIB (9 males, 10 females, 3 unknown sex). Most of individuals were detected at multiple stations in both sites (NNNP: 61%, CIB: 59%) (Table 1).

### 3.2. Density Estimates and Sex Ratio

In NNNP, two SCR models received substantial support (ΔAIC < 2): one allowing both *g*0 and *σ* to vary with sex, and another allowing only *σ* to vary with sex. In CIB, all four competing models performed similarly, with the top-ranked model allowing only *σ* to vary with sex. Sex therefore had a significant influence on leopard movement in both sites but in contrasting directions: in NNNP, males ranged over twice the distance of females (*σ_male_* = 3.4 ± 0.4 km, *σ_female_* = 1.6 ± 0.2 km) while in CIB, females ranged slightly further than males (*σ_male_* = 2.5 ± 0.2 km, *σ_female_* = 3.0 ± 0.4 km) (Table 1). Results of model selection and detailed parameter estimates (e.g., CIs) for both sites are provided in File S4.

**Figure 2:**
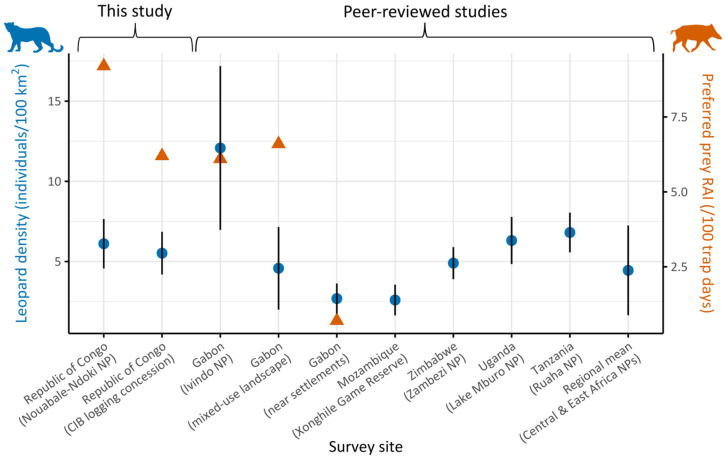
Leopard population density estimates (blue circles, left *Y*-axis) across survey sites in Central and East Africa, organized by country and land-use type (*X*-axis), with error bars showing the standard error for each estimate. Site labels include country names and land-use categories in parentheses (NP = National Park). Estimates are based on this study’s surveys and previously published studies from Gabon [90], Mozambique [91], Zimbabwe [92], Uganda [93], and Tanzania [94]. The final label on the *X*-axis represents the regional mean leopard density across protected areas in Central and East Africa reported in the literature [95]. Relative abundance index (RAI) of red river hog (*Potamochoerus porcus*), a key leopard prey species, was available only for the first five sites and is shown as orange triangles (right *Y*-axis).

We estimated leopard density at 6.11 ± 1.54 individuals per 100 km^2^ in NNNP (95% CIs: 3.76–9.92) and at 5.52 ± 1.33 per 100 km^2^ in CIB (95% CIs: 3.47–8.79) (Figure 2). Analysis of sex ratio parameter (*pmix*) revealed a strong bias towards females in NNNP (77.0 ± 9.8%, *χ*^2^ = 4.2, *p* = 0.04) while no deviation from parity was detected in CIB (*χ*^2^ = 0.3, *p* = 0.6) (Table 1).

Both density estimates had HRCIW values ≤ 50% (NNNP: 50%, CIB: 48%), indicating sufficient precision to detect a 50% population decline, meeting IUCN criteria for assessments under the Endangered status [43].

### 3.3. Spatiotemporal Niche Partitioning

**Figure 3:**
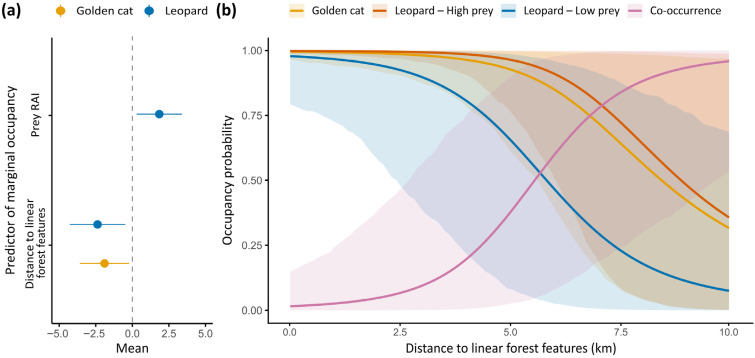
(**a**) Model-estimated coefficients and their associated error for marginal occupancy probability of golden cats and leopards in relation to prey relative abundance index (RAI) and distance to linear forest features (i.e., main river or road). (**b**) Predicted marginal occupancy probability of golden cats, leopards and their co-occurrence probability along a gradient of distance to linear forest features (km). The red curve associated with leopard marginal occupancy refers to stations with high prey abundance (RAI = 14 captures/100 trap days; 85th quantile), and the blue one to stations with low prey abundance (RAI = 3 captures/100 trap days; 15th quantile). Shaded areas represent 95% confidence intervals.

The best-supported model indicated non-random patterns of space use between golden cats and leopards. Both species were more likely to occur in close proximity to linear forest features, while leopard occupancy was also positively associated with prey availability (Figure 3a). Yet, leopards and golden cats were more likely to co-occur at sites further away from these linear forest features, where both species’ marginal occupancy was lowest (Figure 3b). The model including survey site as a predictor of spatial patterns had the highest AIC (File S5), indicating no detectable site-level effect across datasets.

**Figure 4:**
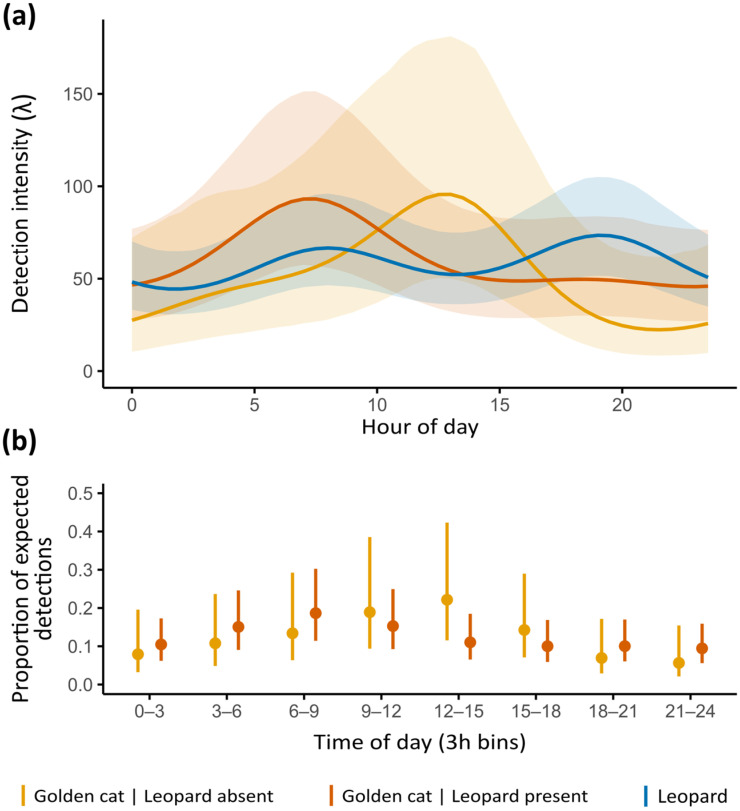
(**a**) Model-predicted daily activity patterns of golden cats at stations with (orange line) and without leopard presence (yellow line), and of leopards (blue line), with shaded areas representing 95% confidence intervals. (**b**) Proportion of expected golden cat detections in the presence (orange lines) and absence of leopards (yellow lines), aggregated in 3 h bins across the 24 h cycle, with error bars representing 95% confidence intervals.

Daily activity patterns from Fourier terms indicated a bimodal, crepuscular activity pattern for leopards (Figure 4a). Golden cats showed a predominantly diurnal peak when leopards were absent. At stations where both felids co-occurred, golden cats appeared to shift peak activity from midday towards dawn, although proportions of expected detections did not differ significantly between stations with and without leopard presence (Figure 4b).

Our results found no significant effect of leopards on golden cat detection (File S5), suggesting no detectable temporal lag effect at the timescale considered. Full model rankings and parameter estimates are provided in File S5.

### 3.4. Trophic Overlap

Due to the difficulty of locating scats from low-density species in dense forest, only 36 samples were collected during camera trap operations. Given the small sample size, the proximity of the two sites, and their similar mammal communities [62], samples were pooled by species. Of the 36 samples processed for genetic identification, 69.4% (*n* = 25) yielded conclusive prey DNA, while 19.4% (*n* = 7) contained DNA that was too degraded, 8.3% (*n* = 3) contained only predator DNA, and 2.8% (*n* = 1) belonged to another carnivore species.

**Figure 5:**
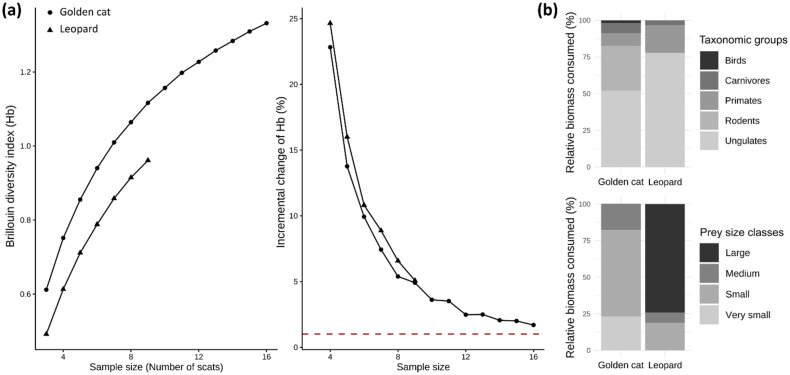
(**a**) Brillouin diversity curves and incremental change curves for golden cat (dots) and leopard (triangles) scat samples. Mean values were estimated by resampling with replacement (10,000 iterations). The horizontal red dotted line marks an incremental change of 1%, below which sampling is considered adequate. (**b**) Composition of taxonomic groups (upper panel) and prey size classes (lower panel) in golden cat and leopard diets. Prey size classes are defined as large (≥30 kg), medium (10–30 kg), small (2–10 kg), and very small (≤2 kg).

Diversity curves did not reach an asymptote for either species, and incremental change in the Brillouin index remained above the 1% adequacy threshold, though golden cat curves approached it after 16 samples (Figure 5a). Results should therefore be interpreted with caution.

A total of 22 prey items were recorded in golden cat scats (*n* = 16), representing 8 different prey species, and 11 prey items in leopard scats (*n* = 9), representing 7 prey species. Mean prey items per scat were 1.38 for golden cats and 1.22 for leopards. Prey composition differed significantly between the two felids (Figure 5b). Based on relative biomass consumed, leopards fed mainly on medium to large ungulates (77.9%), especially red river hogs, with primates making up most of the remainder (18.8%) (Table 2). In contrast, golden cats relied on medium- to small-sized ungulates (52.0%) and rodents (30.6%), occasionally supplementing their diet with small primates (8.6%), small carnivores (6.8%), and birds (1.9%) (Table 2).

Prey size distribution also contrasted strongly (Figure 5b). Leopards specialized on large prey (≥30 kg), driven by their high consumption of red river hogs, while golden cats targeted mostly small (2–10 kg; 58.7%) and very small (≤2 kg; 23.2%) prey, with medium-sized prey comprising 18.1% of their diet (Table 2).

Standardized niche breadth values were 0.27 for leopards and 0.24 for golden cats, indicating dietary specialization in both species. Observed dietary overlap was minimal (Pianka’s index = 0.04), with Peters’ duiker (*Cephalophus callipygus*) being the only prey species shared by both felids.

## 4. Discussion

Our study, conducted in the lowland rainforests of the northern Republic of Congo, is the first to comprehensively examine coexistence patterns between leopards, the region’s apex carnivore, and golden cats, a sympatric mesocarnivore, across spatial, temporal, and trophic dimensions, while also delivering the first robust leopard density estimates for this part of Central Africa. We estimated leopard densities of about 5–6 individuals per 100 km^2^ in both study sites. Regarding coexistence, we found no support for broad-scale spatial segregation between the two felids (H1). However, we detected fine-scale spatial structuring consistent with avoidance of leopards by golden cats, mediated by proximity to linear forest features, with co-occurrence more likely in areas farther from the main river and road. Contrary to our second prediction (H2), we detected no significant temporal partitioning, whereas trophic niches were distinctly segregated (H3). Overall, these results suggest that dietary differentiation is the key axis mitigating intraguild competition and facilitating leopard-golden cat coexistence in this prey-rich system.

### 4.1. Leopard Density

Leopard densities in both study sites were higher than the average reported across protected areas in Central and East Africa [95], highlighting the importance of Congolese forests as a potential stronghold for the species. Yet, our estimates were substantially lower than the estimated leopard density in a remote part of Ivindo National Park in Gabon (*n* = 12.08 ± 5.11 individuals per 100 km^2^; [90]), even though leopards’ prey, including red river hogs and medium-sized duikers (*Cephalophus* spp.), appeared comparable or higher in our sites. This contrast may suggest that anthropogenic pressures play a role in constraining leopard populations below their ecological potential, through persecution (direct and indirect) and indirect fear-mediated effects, as observed elsewhere [92,94], including in neighboring Gabon where leopard density was substantially lower (4.58 ± 2.58 individuals per 100 km^2^; [90]) at the edge of the same national park. Alternatively, part of this observed difference could stem from methodological differences, as Henschel et al. (2011) [90] relied on conventional capture–recapture models, which tend to yield higher density estimates than spatially explicit approaches used in our study. Sex ratio was biased towards females in NNNP, a pattern commonly reported in large carnivores, including in leopard populations [66,96], whereas the balanced structure in CIB might reflect local reduced dispersal [97], which could potentially be associated with behavioral adjustments to rotating logging activities within the concession [96]. Unfortunately, density estimation for golden cats remains challenging in this region due to mostly homogeneous coat patterns among individuals. Future applications of SCR with partial identification [71,98] or methods designed for unmarked populations, such as Camera Trap Distance Sampling (CTDS) [99] or Space-to-Event (STE) models [100], should be investigated [101,102].

### 4.2. Spatial Coexistence

Contrary to our first hypothesis (H1) and to the commonly reported displacement of mesocarnivores by larger carnivores ([103,104]; but see [17]), leopards and golden cats exhibited high spatial overlap at both study sites. Both species showed a higher probability of occurrence near linear forest features, represented by a main river or road cutting through otherwise continuous forest. Such structural elements may offer ecological advantages, including increased prey encounter rates linked to riparian habitats or early-successional vegetation [105,106], facilitated movement along linear corridors, and improved visibility that may enhance hunting efficiency [107]. Linear forest features may also act as natural landmarks regularly used by wild cats to mark their territory boundaries [66]. Leopards, in particular, are known to preferentially use roads when moving through dense habitats [66], a pattern documented in other large felids as well [107,108].

Despite this shared tendency to use areas near linear forest features, co-occurrence between both felids was more likely in forest core areas than near these features, suggesting fine-scale segregation partly consistent with our prediction (H1). This pattern may reflect stronger interference competition in areas with higher visibility—echoing Jenny’s (1996) [66] findings of intensified intraspecific competition among leopards in more open habitats compared to rainforests in Ivory Coast—or species-specific responses to characteristics associated with linear features, such as differences in prey preferences or sensitivity to human disturbance [26,109]. Such local-scale avoidance has been documented in other carnivore guilds [110,111]. For instance, co-occurrence between coyotes (*Canis latrans*) and gray foxes (*Urocyon cinereoargenteus*) was higher in suburban forest fragments in the eastern United States, reflecting shared dependence on these limited habitat refuges [51]. In Tanzania, male leopards and spotted hyenas (*Crocuta crocuta*) were less likely to co-occur near reserve boundaries with low prey occupancy, as hyenas appeared to outcompete leopards that were less tolerant of human disturbance [52].

In our study, co-occurrence between leopards and golden cats was not mediated by the availability of leopards’ preferred prey. However, leopard site use closely tracked that of red river hogs, which is a common spatial pattern linking leopards with large- and medium-sized prey species [112,113].

While interpreting these spatial patterns, it is worth noting that sampling stations located far from linear forest features were underrepresented, limiting inference strength along the full distance gradient, particularly at greater distances. Although this bias cannot account for the observed tendency of both species to occur more frequently near linear features, future studies would benefit from sampling designs explicitly structured to test the effects of such landscape features, with camera trap stations evenly distributed along a broader distance gradient and implemented at larger spatial scales. Replicating such designs across landscapes with varying levels of human access and disturbance would further clarify how ecological responses to linear forest features, such as main rivers and roads, may be mediated by potential indirect influences associated with their role as human access corridors into forest interiors, given the documented sensitivity of both leopards and golden cats to disturbance [64,90].

### 4.3. Temporal Coexistence

At sites where both species co-occurred, their activity patterns overlapped strongly, providing no support for temporal partitioning (H2). Leopards followed a predominantly crepuscular rhythm, consistent with activity patterns elsewhere [112,114], while golden cat activity peaked at dawn but shifted to midday at sites where no leopard has been detected. This suggests that golden cats exploit similar spatiotemporal niches to leopards where the former are present, but expand their activity into a diurnal timeframe in the absence of dominant leopards, possibly reflecting differences in habitat use and the broader prey spectrum typically exploited by mesocarnivores [3,115]. Such coexistence contrasts with our theory-driven expectation of temporal avoidance (H2), a niche dimension often regarded as a key, underestimated mechanism facilitating carnivore coexistence [23,53].

One possible explanation lies in the distinction between reactive and predictive risk responses. Broekhuis et al. (2013) [18] suggested that subordinate carnivores may react to the immediate presence of dominant species rather than anticipating their activity. This pattern has been observed in caracals (*Caracal caracal*), a close relative of golden cat, which exhibited fine-scale spatiotemporal avoidance of leopards in South Africa, with the strength of avoidance increasing under prey scarcity and in more open habitats [22]. Yet, our findings revealed no evidence of such moment-to-moment adjustments, possibly due to the dense vegetation reducing encounter risk and favoring spatiotemporal overlap [66]. We also acknowledge that the limited power of camera traps to capture rapid, fine-scale behavioral adjustments could partly explain this null result [53]. More advanced monitoring approaches are therefore needed to evaluate how responsive behaviors shape carnivore guild interactions [116] and, subsequently, predator-prey dynamics in tropical forest ecosystems [117].

### 4.4. Dietary Segregation

Consistent with our third prediction (H3), we found low dietary overlap between leopards and golden cats, indicating that trophic differentiation is the major mechanism facilitating their coexistence in the northern Congolese forests. While spatial and temporal segregation are often emphasized in carnivore coexistence studies [24,25], our findings highlight the importance of dietary partitioning, an axis likely underestimated due to the scarcity of studies addressing all three dimensions simultaneously [118,119].

Despite the limited sampling effort inherent to rainforest fieldwork and the elusive nature of focal species, our results align with Bahaa-el-din (2015) [120], who similarly reported clear dietary segregation between the two felids using molecular scat analyses. In line with body-size theory [121] and previous research [90,122], leopards primarily consumed prey larger than 20 kg, whereas golden cats relied mainly on small ungulates and rodents (≤5 kg), mirroring patterns observed in other medium-sized felids such as caracals and ocelots (*Leopardus pardalis*) [22,123,124]. While small sample sizes constrain quantitative estimates of niche breadth and Pianka’s overlap, likely underrepresenting rare prey items and narrowing estimated dietary niches, the strong contrast in prey size classes consumed by the two felids, as documented in prior studies [45,120], supports a qualitative pattern of dietary segregation. It is also worth noting that this study represents only the second genetically validated assessment of golden cat diet, thereby providing valuable insights into the trophic ecology of one of the least studied felids worldwide.

Although trophic differentiation emerged as the dominant coexistence mechanism in our study, coexistence is likely mediated by multiple interacting processes. These may include fine-scale avoidance or behavioral adjustments that are difficult to capture with camera trap data alone [125], as well as species-specific responses to prey availability and disturbance. Leopards, in particular, are known for their high ecological plasticity and may shift toward smaller prey species in disturbed, prey-depleted habitats [46,90], potentially increasing dietary overlap with golden cats and intensifying competition when prey diversity or density declines. Understanding how disturbance-driven changes affect niche partitioning and mediate coexistence between the two species should therefore be a key focus of future research. Additionally, expanding molecular diet analyses with larger scat sample sizes would improve estimates of dietary niche breaths and help better account for individual dietary variation, which may bias estimates given its dependence on ecological opportunity and phenotypic traits [126]. Finally, seasonal variation could also influence diet composition, as well as occupancy or activity patterns [127,128], and studies spanning a full annual cycle would therefore provide a more complete understanding of temporal variation in leopard-golden cat coexistence mechanisms. Nevertheless, such seasonal effects are expected to be limited in our study system, as semideciduous forests in Central Africa generally exhibit weak seasonality, and our camera trap data encompass both the main rainy (September-November) and dry (December-February) seasons, likely capturing the greatest potential seasonal variability present.

## 5. Conclusions

Together, our study provides the first robust leopard density estimates for northern Congo and establishes a replicable monitoring framework suitable for long-term ecological assessments across Central African rainforests. By demonstrating that leopards and golden cats displayed selective predation of different-sized prey groups and substantial spatiotemporal overlap, our results strongly suggest that trophic segregation represents a key axis structuring their coexistence in a prey-rich environment. These findings highlight the importance of maintaining prey diversity and minimizing anthropogenic disturbance to ensure the persistence of both species, their coexistence mechanisms, and the ecological functions they support. More broadly, our results underscore the need to adopt multidimensional approaches that integrate spatial, temporal, and trophic perspectives when examining intraguild interactions. In a context where tropical carnivore communities face mounting pressures from habitat degradation, prey depletion, and snaring [39,129], such integrative frameworks are essential for predicting and mitigating shifts in carnivore coexistence and community structure.

## Figures and Tables

**Table 1:** Survey effort and summary results for golden cat and leopard detections, and leopard population density (with associated parameter estimates) for Nouabalé-Ndoki National Park and the ‘Congolaise Industrielle des Bois’ logging concession in the northern Republic of Congo, 2022–2023.

General Results	NNNP	CIB
Survey duration (days)	147	217
Stations	63	62
w/leopard and golden cat	37 (58.7%)	40 (63.5%)
w/golden cat only	13 (20.6%)	15 (23.8%)
w/leopard only	8 (12.7%)	5 (7.9%)
no leopard no golden cat	5 (7.9%)	2 (3.1%)
Trap nights (station-level)	8246	11,560
Trap nights (camera-level)	15,535	19,381
**Golden cat**		
Detections ^a^	170	197
Independent captures ^b^	152	175
Capture rate (/100 trap nights) ^c^	1.8	1.5
Naïve occupancy ^d^	0.79	0.89
**Leopard**		
Detections ^a^	230	226
Independent captures ^b^	186	179
Capture rate (/100 trap nights) ^c^	2.3	1.5
Naïve occupancy ^d^	0.71	0.73
SCR analyses—leopards		
Survey duration (days)	110	198
Trap nights (camera-level)	12,892	18,633
Capture events	162	168
Mask size (km)	14	14
Individuals recorded ^e^	18 (7 ♂, 7 ♀, 4NA)	22 (9 ♂, 10 ♀, 3NA)
Spatial recapture rate ^f^	0.61	0.59
*g*0 ^g^ (♀)	0.009 ± 0.002	0.007 ± 0.001
*g*0 ^g^ (♂)	0.012 ± 0.002	0.007 ± 0.002
*σ* ^h^ (♀)	1570 ± 160	2965 ± 383
*σ* ^h^ (♂)	3406 ± 381	2480 ± 201
Density ^i^: Mean ± SE	6.11 ± 1.54	5.52 ± 1.33
Density ^i^: 95% CI	3.76–9.92	3.47–8.79
Sex ratio ^j^	≠1:1 (*p* = 0.04)	=1:1 (*p* = 0.57)
HRCIW ^k^ (%)	50	48

^a^ Photographs taken > 1 min apart. ^b^ Photographs taken >30 min apart. ^c^ Capture rates were calculated using station-level trap nights to account for non-independence of paired cameras at the same station. ^d^ Proportions of stations occupied. ^e^ Based on both flank captures. Includes only identifiable captures. ^f^ Percentage of identified individuals captured at more than one station during the survey period. ^g^ Capture probability at home range center and associated SE. ^h^ Spatial movement parameter and associated SE (m). ^i^ Population density defined as the number of individuals per 100 km^2^. ^j^ Statistical results indicating whether the sex ratio is significantly different from 1:1. ^k^ Half relative confidence interval width, providing a measure of the magnitude of population change that an estimate has a reasonable probability of detecting [74].

**Table 2:** Diet composition of leopards and golden cats in the forests of the northern Republic of Congo, expressed as frequency of occurrence (FO), corrected frequency of occurrence (CFO, adjusted for multiple prey items per scat), and relative biomass consumed (RBC).

		Leopard (*n* = 9) ^a^			Golden Cat (*n* = 16) ^a^		
Prey Species	ABM ^b^ (kg)	FO (%)	CFO (%)	RBC (%)	FO (%)	CFO (%)	RBC (%)
Ungulates		**77.8**	**70.4**	**77.9**	**50.0**	**37.5**	**52.0**
*Cephalophus callipygus*	20.8	11.1	3.7	3.8	12.5	9.4	13.6
*Cephalophus* sp.	19.2 ^c^				6.3	3.1	4.5
*Cephalophus sylvicultor*	69.2	11.1	11.1	12.4			
*Philantomba monticola*	5.1				**31.3**	**25.0**	**33.9**
*Potamochoerus porcus*	53.3	**55.6**	**55.6**	**61.8**			
Primates		**33.3**	**25.9**	18.8	6.3	6.3	8.6
*Cercopithecus nictitans*	5.4				6.3	6.3	8.6
*Cercopithecus pogonias*	3.7	11.1	3.7	2.1			
*Colobus guereza*	8.4	11.1	11.1	8.5			
*Lophocebus albigena*	7.7	11.1	11.1	8.2			
Rodents					**62.5**	**46.9**	**30.6**
*Atherurus africanus*	3				6.3	6.3	7.5
*Cricetomys emini*	0.9				12.5	9.4	7.1
*Murinae*	0.25 ^c^				**43.8**	**31.3**	16.0
Carnivores		11.1	3.7	3.3	6.3	6.3	6.8
*Civettictis civetta*	12.6	11.1	3.7	3.3			
*Genetta servalina*	2.3				6.3	6.3	6.8
Birds	0.5 ^c^	0.0	0.0	0.0	6.3	3.1	1.9

Note: Boldface indicates the most frequently consumed (≥25%) prey items for both felids. ^a^ Number of scats used in the analyses. ^b^ ABM = Average Body Mass, averaged by taking mean body mass for adult males and females from Kingdon (2015) [84]. ^c^ ABM averaged from the most likely species within the genus, family, or class assigned during taxonomic identification.

## Data Availability

Data are not publicly available due to conservation concerns. Both the African golden cat and leopard are Vulnerable species, and sharing their locations could increase poaching risks. Data may be made available from the corresponding author upon reasonable request and with appropriate safeguards.

## Supplementary Materials

The following supporting information can be downloaded at: https://www.mdpi.com/article/10.3390/ani16020190/s1. File S1: List of the species detected and summary of their detection details in our two sites in Central African forests; File S2: Distribution of camera trap stations along the gradient of distance to linear forest features; File S3: Taxonomic assignment details and formulas for diet analyses; File S4: Results of SCR model selection and detailed parameter estimates; File S5: Results of occupancy model selection and detailed parameter estimates.
