# Supplementary material for: Density and Coexistence Patterns of an Apex Carnivore (Panthera pardus) and a Mesocarnivore (Caracal aurata) in Northern Congo Forests
Source: Animals (Basel). 2026 Jan 8;16(2):190. doi: 10.3390/ani16020190 (PMC12837921; doi:10.3390/ani16020190)
Supplement: Supplementary file 1 [file animals-16-00190-s001.zip › File S1_Summary_detections.pdf]

File S1. Summary of detections, RAI (relative abundance index; the number of independent capture events per 100 trap days), number of stations occupied, and naïve occupancy (proportions of stations occupied) for each terrestrial and semi-terrestrial mammal and ground bird species (>0.5kg) surveyed in our two study sites in Central African forests, over August-February 2022, during 8246 and 11478 camera days respectively.

Order	Family	Species	Noubalé-Ndoki National Park (NNNP)				Congolaise Industrielle des Bois (CIB)				IUCN
			n	RAI	Stations occupied	Naïve occupancy	n	RAI	Stations occupied	Naïve occupancy	
**TERRESTRIAL & SEMI-TERRESTRIAL MAMMALS**											
**Artiodactyla**											
	**Bovidae**	*Cephalophus callipygus*	6817	82.7	63	1.00	6204	54.1	62	1.00	LC
		Cephalophus dorsalis	558	6.8	55	0.87	513	4.5	61	0.98	NT
		Cephalophus leucogaster	320	3.9	43	0.68	19	0.2	12	0.19	NT
		Cephalophus nigrifrons	14	0.2	6	0.10	-	-	-	-	LC
		Cephalophus silvicultor	1727	20.9	62	0.98	1405	12.2	62	1.00	NT
		Philantomba monticola	12043	146.0	63	1.00	13565	118.2	62	1.00	LC
		Syncerus caffer nanus	41	0.5	22	0.35	119	1.0	25	0.40	NT
		Tragelaphus eurycerus	47	0.6	10	0.16	191	1.7	40	0.65	NT
		Tragelaphus spekii	-	-	-	-	1	0.0	1	0.02	LC
	**Suidae**	*Hylochoerus meinertzhageni*	12	0.1	8	0.13	51	0.4	24	0.39	LC
		Potamochoerus porcus	755	9.2	61	0.97	708	6.2	59	0.95	LC
	**Tragulidae**	*Hyemoschus aquaticus*	44	0.5	10	0.16	25	0.2	3	0.05	LC
**Carnivora**											
	**Felidae**	*Caracal aurata*	130	1.6	49	0.78	159	1.4	53	0.85	**VU**
		Panthera pardus	166	2.0	43	0.68	158	1.4	43	0.69	**VU**
	**Herpestidae**	*Bdeogale nigripes*	69	0.8	33	0.52	272	2.4	53	0.85	LC
		Crossarchus platycephalus	3	0.04	3	0.05	68	0.6	24	0.39	LC
		Mongooses	187	2.3	42	0.67	612	5.3	59	0.95	LC

	**Mustelidae**	*Aonyx congicus*	-	-	-	-	1	0.0	1	0.02	NT
		Mellivora capensis	36	0.4	23	0.37	7	0.1	6	0.10	LC
	**Nandiniidae**	*Nandinia binotata*	62	0.8	36	0.57	94	0.8	36	0.58	LC
	**Viverridae**	*Civettictis civetta*	40	0.5	11	0.17	4	0.0	2	0.03	LC
		Large spotted genets	360	4.4	60	0.95	514	4.5	59	0.95	**VU**-LC
**Hyracoidea**											
	**Procaviidae**	*Dendrohyrax dorsalis*	-	-	-	-	1	0.0	1	0.02	LC
**Pholidota**											
	**Manidae**	Small pangolins	18	0.2	15	0.24	29	0.3	23	0.37	**EN-VU**
		Smutsia gigantea	32	0.4	15	0.24	9	0.1	6	0.10	**EN**
**Primates**											
	**Cercopithecidae**	*Cercocebus agilis*	58	0.7	24	0.38	166	1.4	44	0.71	LC
	**Hominidae**	*Gorilla gorilla*	491	6.0	62	0.98	556	4.8	61	0.98	**CR**
		Pan troglodytes	675	8.2	60	0.95	766	6.7	60	0.97	**EN**
**Proboscidea**											
	**Elephantidae**	*Loxodonta cyclotis*	1996	24.2	63	1.00	2502	21.8	62	1.00	**CR**
**Rodentia**											
	**Hystricidae**	*Atherurus africanus*	146	1.8	26	0.41	451	3.9	49	0.79	LC
	**Nesomyidae**	*Cricetomys emini*	122	1.5	35	0.56	291	2.5	44	0.71	LC
	**Sciuridae**	Forest squirrels	755	9.2	61	0.97	658	5.7	49	0.79	-
**Tubulidentata**											
	**Orycteropodidae**	*Orycteropus afer*	68	0.8	34	0.54	56	0.5	24	0.39	LC
**GROUND BIRDS**											
**Galliformes**											
	**Numididae**	*Agelastes niger*	117	1.4	31	0.5	101	0.9	23	0.4	LC
		Guttera plumifera	142	1.7	43	0.7	393	3.4	58	0.9	LC
**Gruiformes**											
	**Rallidae**	*Himantornis haematopus*	55	0.7	16	0.3	100	0.9	29	0.5	LC
