# Supplementary material for: Density and Coexistence Patterns of an Apex Carnivore (Panthera pardus) and a Mesocarnivore (Caracal aurata) in Northern Congo Forests
Source: Animals (Basel). 2026 Jan 8;16(2):190. doi: 10.3390/ani16020190 (PMC12837921; doi:10.3390/ani16020190)

File S2. Distribution of the 63 and 62 stations in the Nouabale-Ndoki National Park (NNNP) and the logging concession *Congolaise Industrielle des Bois* (CIB), respectively, along the gradient of linear forest features (*i.e.*, main river or road).

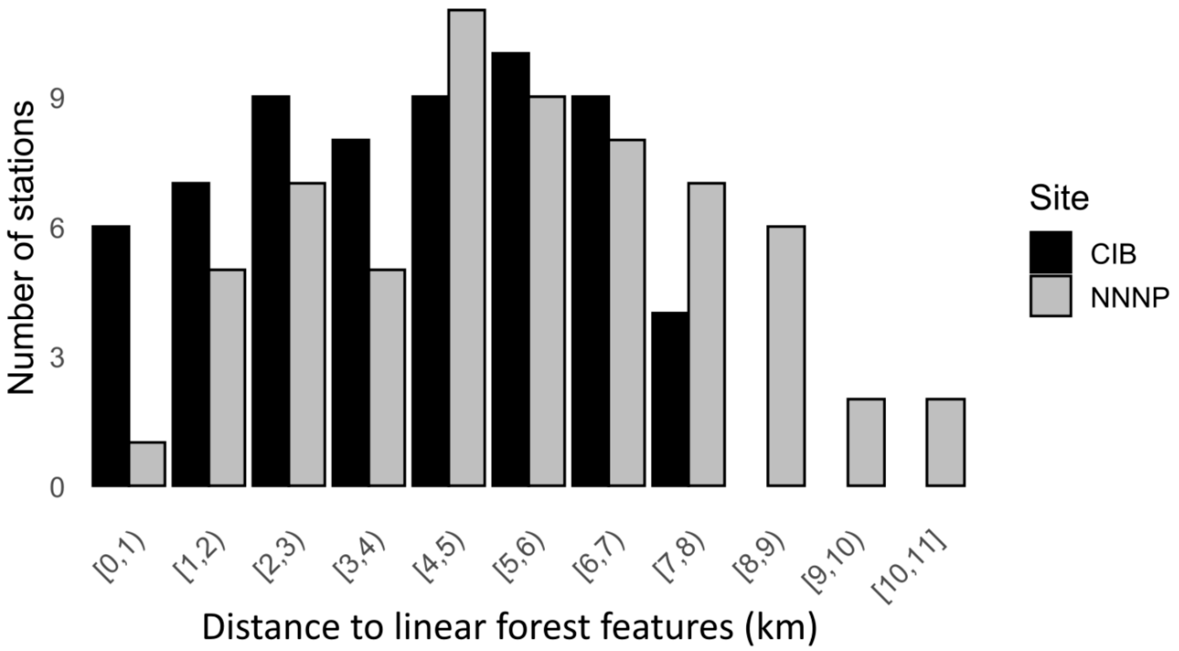

Supplement: Supplementary file 1 [file animals-16-00190-s001.zip › File S2_Dist_linear_forest_features.pdf]
