# Supplementary material for: Density and Coexistence Patterns of an Apex Carnivore (Panthera pardus) and a Mesocarnivore (Caracal aurata) in Northern Congo Forests
Source: Animals (Basel). 2026 Jan 8;16(2):190. doi: 10.3390/ani16020190 (PMC12837921; doi:10.3390/ani16020190)
Supplement: Supplementary file 1 [file animals-16-00190-s001.zip › File S3_molecular_analyses.pdf]

Taxonomic assignment

1) *BOLD species-level assignment*: OTUs with ≥99% query cover in BOLD were assigned to species.
2) *Secondary database check*: OTUs with 90-98% query cover in BOLD and >0.01% of total reads were searched in NCBI GenBank for higher-rank assignment.
3) *Consensus rank assignment*: If NCBI hits had ≥99% query cover, the shared genus or family between BOLD and NCBI was assigned (*e.g.*, *Cephalophus sp*., Murinae).
4) *Species-specific exception*: For African golden cat (*Caracal aurata*), NCBI hits matched *Profelis aurata* (99% cover, 95.5% identity) and *Caracal caracal* (99% cover, 93.2% identity). As *Caracal aurata* is the only non-leopard felid in the study area, it was assigned to species.
5) *Geographical verification*: All species distributions were checked using IUCN (2025) and local records. One mismatch (*Colobus polykomos*) was reassigned to *Colobus guereza*, the only *Colobus sp.* in the survey area.

Diet analyses

Frequency of occurrence metrics

We used three occurrence-based measures:

- FO = percentage of scats containing a given prey category;
- CFO = FO corrected for multiple prey per scat by assigning each item a fractional value summing to one per scat sample (*e.g.*, two items scored 0.5 each);
- RFO = proportion of a prey category relative to total prey occurrences across all samples.

Ingested biomass estimates

Biomass consumed was estimated with the asymptotic regression model of Chakrabarti et al. (2016) for obligate carnivores:

$$Y = \left(0.033 - 0.025\, e^{-4.284\left(\frac{X}{PBM}\right)}\right) x\ PBM$$

where Y = mass of prey consumed per collectable scat (kg), X = mean prey body mass (kg), and PBM = predator body mass (kg).

Trophic niche breadth

Levin's niche breadth(Levins, 1968) was calculated as:

$$B = \frac{1}{\sum P_i^2}$$

where P_i is the CFO of prey category i. Standardized niche breath was then:

$$BA = \frac{B - 1}{n - 1}$$

where n = number of prey categories. BA ranges from 0 (specialist) to 1 (generalist).

Trophic niche overlap

Dietary overlap between leopards and golden cats was calculated using Pianka's index (Pianka, 1973):

$$O_{jk} = \frac{\sum P_{ij} P_{ik}}{\sqrt{\sum P_{ij}^2 \sum P_{ik}^2}}$$

Where P_{ij} and P_{ik} are the proportions of prey category i in the diets of predators j and k. The index ranges from 0 (no overlap) and 1 (complete overlap).

Sampling adequacy

Sampling adequacy was assessed using the Brillouin index (Brillouin, 1962):

$$H_b = \frac{\ln N! - \sum \ln n_i!}{N}$$

Where N = total prey items, n_i = number of items in category i. Curves generated via bootstrapping (10 000 resamples) across increasing sample sizes. Adequacy was considered reached when the diversity curve approached an asymptote and incremental change fell below 1% (Hass, 2009).

References

Brillouin, L. (1962). *Science and information theory* (2nd ed). Academic Press.

Hass, C. C. (2009). Competition and coexistence in sympatric bobcats and pumas. *Journal of Zoology, 278*(3), 174–180. https://doi.org/10.1111/J.1469-7998.2009.00565.X

IUCN. (2025). *The IUCN Red List of Threatened Species. Version 2025-1.* http://www.iucnredlist.org/

Levins, R. (1968). *Evolution in changing environments: some theoretical explorations.* Princeton University Press.

Pianka, E. R. (1973). *The structure of lizard communities* (Vol. 4). Annual Review of Ecology and Systematics.
